# Speed of Sound Measurements of Select Ternary Refrigerant Mixtures and Predictions Using Constituent Binary Interaction Parameters

**DOI:** 10.1007/s10765-025-03608-3

**Published:** 2025-09

**Authors:** Karim S. Al-Barghouti, Katrina N. Avery, Ian H. Bell, Aaron J. Rowane

**Affiliations:** 1 Applied Chemicals and Materials Division, National Institute of Standards and Technology, Boulder, CO 80305, USA

**Keywords:** Speed of sound, Ternary mixtures, Pulse-echo, Equation of state, Refrigerants

## Abstract

The speeds of sound of ternary refrigerant mixtures, namely, R-444A (difluoromethane (R-3 2)/1,1 -difluoroethane (R-15 2a)/*trans* −1,3,3,3-tetrafluoropropene (R-1234ze(E)) with respective mass fractions of 0.1194/0.0519/0.8287), R-457B (R-32/2,3,3,3-tetrafluoropropene (R-1234yf)/R-152a with respective mass fractions of 0.3489/0.5495/0.1016), and R-407C (R-32/pentafluoroethane (R-125)/R-152a with respective mass fractions of 0.5178/0.2480/0.2342), were measured using a dual-path pulse-echo technique at temperatures ranging between 230 K and 345 K and pressures between 0.14 MPa and 30 MPa. The standard uncertainties in temperature and pressure were 5 mK and 0.014 MPa, respectively. The average combined expanded uncertainty for all speed of sound data was 0.07%. Greater uncertainties were encountered as the system approached the critical regions where the speed of sound is more sensitive to changes in pressure. The experimental speed of sound data was used to assess the predictive capabilities of default REFPROP v10.0 mixture models with binary interaction parameters fit using mainly vapor-liquid equilibria and/or density data. We quantify the improvements for ternary mixture predictions when using updated binary interaction parameters that included speed of sound data in the fitting procedure. Reductions of 1.64%, 1.50%, and 0.11% in the average absolute deviations for R-444A, R-457B, and a R-125/1234yf/152a (0.3521/0.5465/0.1014 mass composition) mixture, respectively, are obtained with the updated binary interaction parameters. Further improvements to the mixture models could be made by refitting both the pure component equations of state and interaction parameters of certain hydrofluorocarbon binary pairs.

## 1 Introduction

Hydrofluorocarbon gases are widely used as refrigerants, propellants, and foam blowing agents among other applications. Blends of hydrofluorocarbons (HFCs) and hydrofluoroolefins (HFOs) are being investigated as replacements for many HFCs and hydrochlorofluorocarbons (HCFCs) in several applications including automotive air-conditioning and household refrigerators. High-accuracy thermodynamic and transport data of these pure components and mixtures are imperative for various design considerations.

In previous studies, we have measured vapor-liquid equlibrium (VLE), density, and speed of sound of several binary mixtures containing HFCs and HFOs. These studies included blends of HFOs 2,3,3,3-tetrafluoropropene (R-1234yf) and *trans* - 1,3,3,3-tetrafluoropropene (R-1234ze(E)) with HFCs 1,1,1,2-tetrafluoroethane (R-134a), difluoromethane (R-32), pentafluoroethane (R-125), 1,1-difluoroethane (R-152a), and 1,1,1,2,3,3,3-heptafluoropropane (R-227ea) [1–7]. However, to meet the demands of various refrigeration applications, industry has identified blends with up to six components as classified in ASHRAE Standard 34 [8]. It is not feasible to measure thermodynamic properties of every possible blend to screen for suitable multicomponent mixtures for every application. Therefore, models that treat multicomponent mixtures in terms of their constituent binary pairs are often used to predict the thermodyanmic properties of these blends and assess their applicability in a target application. The more reasonable approach is to only perform measurements on blends that show promise for a specific application to ensure that the multifluid models accurately represent important thermodynamic properties.

The thermodynamic properties for common pure HFCs and HFOs have been measured by our group and others to develop reference Helmholtz-energy-explicit equations of state (EoS) for several pure refrigerants [9–12]. These pure fluid EoS and data for binary mixtures were used to obtain binary interaction parameters for mixture models, which are defined by Span [13] as multifluid models. Such models are implemented in REFPROP [12], but many were fit only to VLE and/or density data. In contrast, Bell recently fit bubble point, density, and speed of sound data of binary mixtures to multifluid models to obtain binary interaction parameters, and these models were shown to accurately represent the mixtures’ thermodynamic properties [14, 15].

In this work, we investigate how well the present pure fluid EoS and fitted binary interaction parameters alone can represent the speed of sound of ternary mixtures with no additional fitting. We chose three ternary mixtures from ASHRAE 34 for the purpose of this study, which are R-407C (R-32/125/134a with respective mass fractions of 0.5178/0.2480/0.2342), R-444A [R-32/152a/1234ze(E) (0.1194/0.0519/0.8287)], and R-457B [R-32/1234yf/152a (0.3489/0.5495/0.1016)]. Additionally, we measured the speed of sound of a ternary mixture similar to R-457B but replaced R-32 with R-125 to have the blend R-125/1234yf/152a (0.3521/0.5465/0.1014) as an additional test to the multifluid model’s ability to represent the speed of sound of ternary mixtures. This blend will be referred to as Ternary 4 throughout this work. This study does not focus on experimental investigations of binary pairs, but the interested reader is directed to the work of Bobbo et al. [16], Fedele et al. [17], and Bell et al. [18] for available thermodynamic data on refrigerant binary pairs.

The mixtures in this work were selected based on several practical considerations and industrial relevance. R-407C, purely an HFC blend, was proposed as a substitute for chlorodifluoromethane (R-22) as they both have similar thermophysical properties [19]. Although R-407C is being phased out, it provides an important baseline to validate our technique for mixtures with more than two components [20]. R-444A and R-457B have been proposed as potential substitutes for several HFC refrigerants [21, 22]. R-457B is a promising replacement for R-22 in high ambient temperature applications [23], and R-444A has been proposed as a replacement for R-134a in automobile air-conditioning (AC) systems and household refrigerators [24, 25]. R-444A may replace R-1234yf in automotive AC systems as well [24]. The measured speed of sound data for each ternary mixture was compared to both the default REFPROP v10.0 models [26] and models with binary interaction parameters updated by Bell [14, 15]. It is important to note that prior to the studies by Rowane and Perkins [2, 6], there was an extreme paucity of speed of sound data for blends of HFC and HFO refrigerants. Therefore, in contrast to the recent models of Bell [14, 15], many of the REF-PROP v10.0 default models did not include speed of sound data in the fitting routine. This work assesses the predictive accuracy obtainable using default REF-PROP v10.0 models and quantifies the improvements achievable with the updated interaction parameters that explicitly fit speed of sound data.

## 2 Experimental Section

### 2.1 Materials and Methods

Table 1 lists the refrigerants used in this study along with their short names, CAS numbers, molar mass, source, and purity. The component purities are those reported by the supplier. All pure refrigerants were degassed using a freeze-pump-thaw method prior to preparing the mixtures [1].

Ternary refrigerant mixtures were prepared in the vapor phase, and the composition was determined gravimetrically using the double substitution method [27]. Standard deviations of four weighing cycles were between 0.0007 g and 0.0035 g, which translate to an uncertainty contribution from the gravimetric preparation of 1.1∙10^−5^ and 1.5∙10^−5^ mass fraction, respectively. Such uncertainties can be especially relevant when comparing experimental thermodynamic data to model performance where compositions are assumed to be exact. One must consider the range of values possible for a property given varying compositions with a sum of unity within the bounds of the uncertainty limits. However, the low mass fraction uncertainties obtained here are expected to yield negligible contributions to the evaluated model deviations relative to the contributions inherent to the pure fluid EoS and binary interaction parameters.

To account for sample impurities in our composition uncertainty analysis, the product of the sample mole fraction and the impurity was added in quadrature with the uncertainty determined from gravimetric preparation analogous to our previous study [6]. Additional contributions that are not easily measured include contamination of the sample cylinder, expansion of the cylinder when filling, preferential adsorption of a mixture constituent [28] onto the cylinder walls, valves, or tubing. These uncertainties were estimated to be 0.00010 mass fraction. Table 2 lists the mixture components, ASHRAE designations, mass fractions of the components, and the mass fraction uncertainty.

### 2.2 Dual-Path Pulse-Echo Technique and Instrument

The dual-path pulse-echo instrument used in this study is the same as that described by McLinden and Perkins [29]; therefore, only the key features of the instrument are summarized here. The dual-path pulse-echo technique involves applying an input voltage pulse consisting of 10 sinusoids to a quartz crystal immersed in the fluid sample, which causes the transducer to emit sound bursts from its opposing sides. The transducer used in our instrument was a quartz crystal disk with an 8 MHz resonant frequency. This quartz crystal disk was secured between two ceramic spacers that defined the short and long path lengths, which were approximately 12 mm and 30 mm in length, respectively. On opposing ends of the spacers were brass reflectors. These components were contained within a support tube, which was contained within a stainless-steel pressure vessel with a pressure rating of 93 MPa.

The sound bursts emitted from the transducer traverse the sample of interest along the short and long path lengths. The sound bursts are reflected at the end of each path back to the quartz crystal which also acts as a receiver and is connected to a three-stage amplifier. The signal from the amplifier is recorded with an oscilloscope. The governing equation for the dual-path pulse-echo instrument is defined by

(1) $$w\;=\;\frac{2\left(L_{\mathrm{long}}\;-\;L_{\mathrm{short}}\right)}{\Delta t},$$

where $L_{\mathrm{long}}$ and $L_{\mathrm{short}}$ are the path lengths and Δt is the time difference between the return of the two echo signals.

The signal recorded by the oscilloscope is echoes that are similar in shape but have differing amplitudes. Δt was obtained by superimposing these echoes while simultaneously adjusting the amplitude of the long path echo to match that of the short path. This procedure involved varying a guess value of Δt then plotting the variance between the echo datasets. This allowed us to select the minimum of the variance and obtain the optimum value of Δt. This regression procedure is described in detail by McLinden and Perkins [29]. In this study, independently recorded short and long path data were used in the analysis procedure to minimize the uncertainty in Δt [30]. Typically, 256 echo signals were averaged, but at state points where weaker echo signals were encountered, up to 4096 echoes were averaged to increase the signal-to-noise ratio.

The standard uncertainty of the pressure measurement was 0.014 MPa. The pressure vessel was suspended in a thermostatted bath, with a temperature range of 228 K to 423 K, to control its temperature. Adjacent to the pressure vessel was a standard resistance platinum thermometer to measure the temperature. The standard platinum resistance thermometer was connected to a bridge and ratioed to a standard resistor, which was calibrated against fixed-point cells from 234.316 K to 505.078 K. The standard uncertainty of the temperature measurement was 0.005 K.

The combined expanded uncertainty in the speed of sound measurements, *U*_c_(*w*), determined with a coverage factor of *k* = 2, ranged from 0.037% to 0.41% of the measured speed of sound value. The details of the uncertainty analysis are provided elsewhere [29]. The uncertainty analysis included contributions from the composition, temperature, pressure, time delay between echo arrivals, and path length calibration. The measurements were also corrected for diffraction effects, which had an uncertainty contribution on the order of 0.001% in speed of sound. The greatest contribution in the speed of sound uncertainty was found to be that from the pressure measurements. This is because as the speed of sound approaches a mixture critical point, the rate of change in the speed of sound with pressure dramatically increases. The mixture critical temperatures were estimated to be 359.23 K, 378.47 K, 357.1 K, and 361.39 K for R-407C, R-444A, R-457B, and the Ternary 4 blends, respectively, using the most up to date mixture models described in Sect. 3.3.

## 3 Experimental Results

In the following three sections, we describe the experimental data obtained in this study and compare it to mixture models included in REFPROP version 10.0 [26], and improved models incorporating binary interaction parameters reported by Bell [14, 15].

### 3.1 Measured Data

**Fig. 1:**
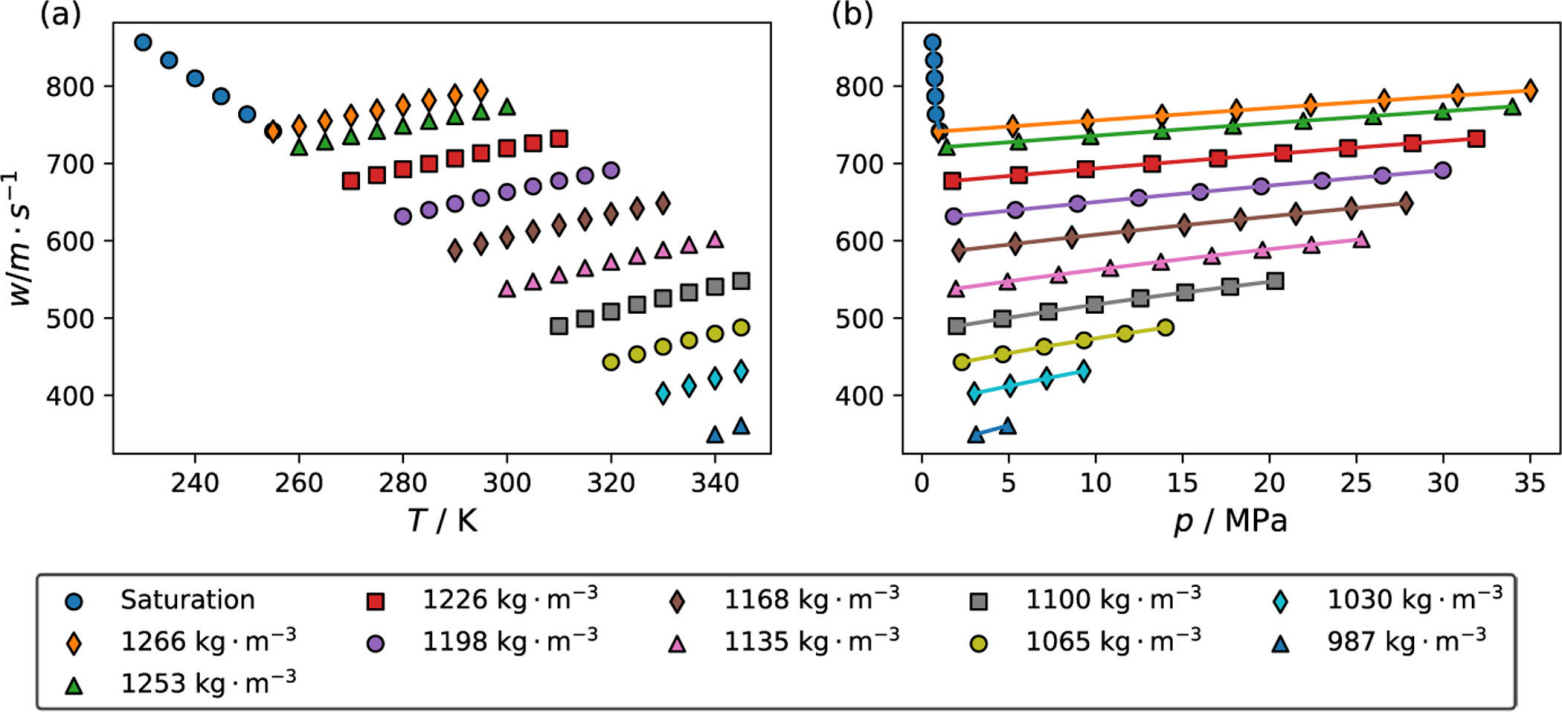
The effect of (a) temperature and (b) pressure on the speed of sound of R-444A. Different symbols in the legend represent the different pseudo-isochores measured. Lines are visual guides indicating data from the same isochore. The densities for each isochore are average densities across all state points for a given isochore, which were calculated using the latest EoS [31–33] for each component and the updated binary interaction parameters reported by Bell [14, 15]

Figure 1a and b show the measured speed of sound for R-444A as a function of temperature and pressure, respectively. All measurements were conducted along pseudo-isochores. The different symbols in the plot represent the different pseudo-isochores measured. Data plots for the other mixtures are omitted here for brevity, but are included in Figures S1, S2, and S3 of the supplementary information file. Tables 3, 4, 5, and 6 list the experimental data for R-407C, R-444A, R-457B, and the Ternary 4 blend. Each data table lists an average of up to twelve replicate speed of sound measurements, the corresponding temperature and pressure for the measured speed of sound data point, and the combined expanded relative state point uncertainty (*U*_r_(*w*)).

### 3.2 Comparison to Literature Data

**Fig. 2:**
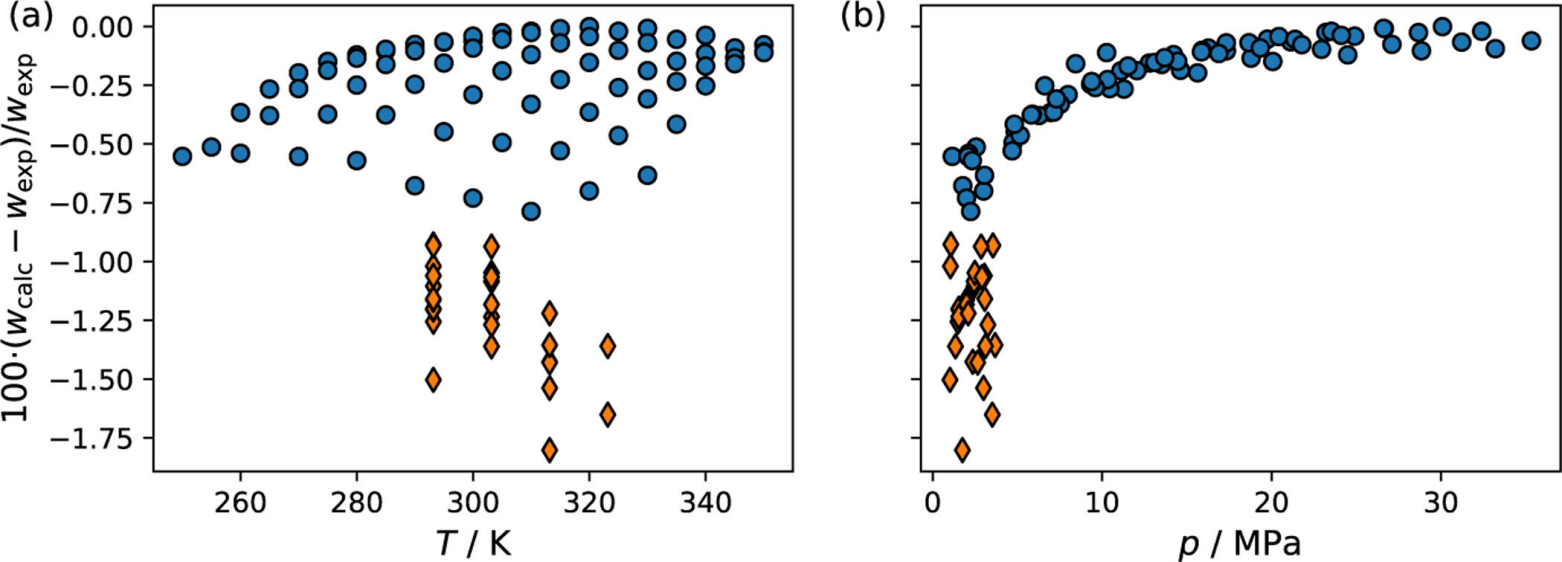
Comparison of the experimental speed of sound data for R-407C reported in this study 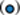 and those reported by Komarov and Stankus [34] 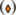 to the mixture model for R-407C included in REFPROP v10.0 [26]

The only liquid phase speed of sound data available for comparison in the literature is that reported by Komarov and Stankus for R-407C [34]. No studies report speed of sound data for the R-444A, R-457B, or Ternary 4 mixtures. Figure 2a and b are deviation plots comparing the R-407C data reported in this study and those of Komarov and Stankus to the current mixture model for R-407C included in REF-PROP v10.0 as a function of temperature and pressure, respectively.

The performance of the models compared to experimental data throughout this work is characterized by the absolute average deviation ($\Delta_{\mathrm{AAD}}$), maximum deviation ($\Delta_{\max}$), and mean bias error ($\Delta_{\mathrm{bias}}$) defined by

(2) $${△}_{\mathrm{AAD}}\;=\;100\;•\;\frac{1}{N}\;\sum_{i=0}^{N}\left|\frac{w_{i,exp}\;-\;w_{i,\mathrm{calc}}}{w_{i,\exp}}\right|$$

(3) $${△}_{\max}\;=\;\max\;\left(100\;•\;\left|\frac{w_{i,exp}\;-\;w_{i,\mathrm{calc}}}{w_{i,\exp}}\right|\right)$$

(4) $$\Delta_{bias}\;=\;100\;•\;\frac{1}{N}\;\sum_{i=0}^{N}\frac{\left(w_{i,\mathrm{calc}}\;-\;w_{i,\exp}\right)}{w_{i,\exp}}$$

where $w_{i,\exp}$ represents an experimentally measured speed of sound value, $w_{i,\mathrm{calc}}$ represents a speed of sound calculated using the applicable multifluid model, and *N* is the number of data points that were included in the average.

Figure 2a shows a discrepancy between the data measured here and that of Komarov and Stankus. However, Fig. 2b shows that the mixture model systematically deviates from both datasets as the pressure approaches the mixture bubble point. This comparison shows that adjustments to the R-407C mixture model may be warranted. It is important to note that the R-407C model includes the EoS of Tillner-Roth and Yokozeki for R-32 [32], which was shown by Rowane et al. [35] and Bell [15] to have deficiencies in reproducing speed of sound data. An improved reference EoS for R-32 and subsequent refitting of the binary interaction parameters for R-32/125 and R-32/134a may improve this mixture model.

### 3.3 Comparison of REFPROP Version 10.0 Multi-fluid Models to Updated Multi-fluid Models

The data reported in this study for R-444A, R-457B, and Ternary 4 were compared to both the multifluid models available in REFPROP v10.0 and the models with updated binary interaction parameters. R-407C does not include any HFOs, and hence, the data were compared only to the default REFPROP v10.0 models (shown in Fig. 2). Table 7 lists the source for each pure fluid equation of state used, the source for the binary interaction parameters for each mixture, the absolute average deviation, and the maximum deviation. The default REFPROP models and the updated models use the same pure fluid EoS except for R-1234yf where the former employs the EoS of Richter et al. [36] and the latter uses the more recent EoS of Lemmon and Akasaka [37]. The results in Table 7 show that the updated binary interaction parameters greatly improve the performance of the R-444A and R-457B mixtures where the $\Delta_{\mathrm{AAD}}$ is reduced by more than a factor of 7 and 4, respectively. This is not surprising since the updated models explicitly used speed of sound data in the fitting procedure. The prediction improvements for the Ternary 4 mixture are less drastic, only improving by 0.11% when the updated binary interaction parameters are used. However, it should be noted that the default REFPROP v10.0 models for R-32/152a and R-125/1234yf do not have a corresponding journal publication, and the nature of the fitted data is unclear.

**Fig. 3:**
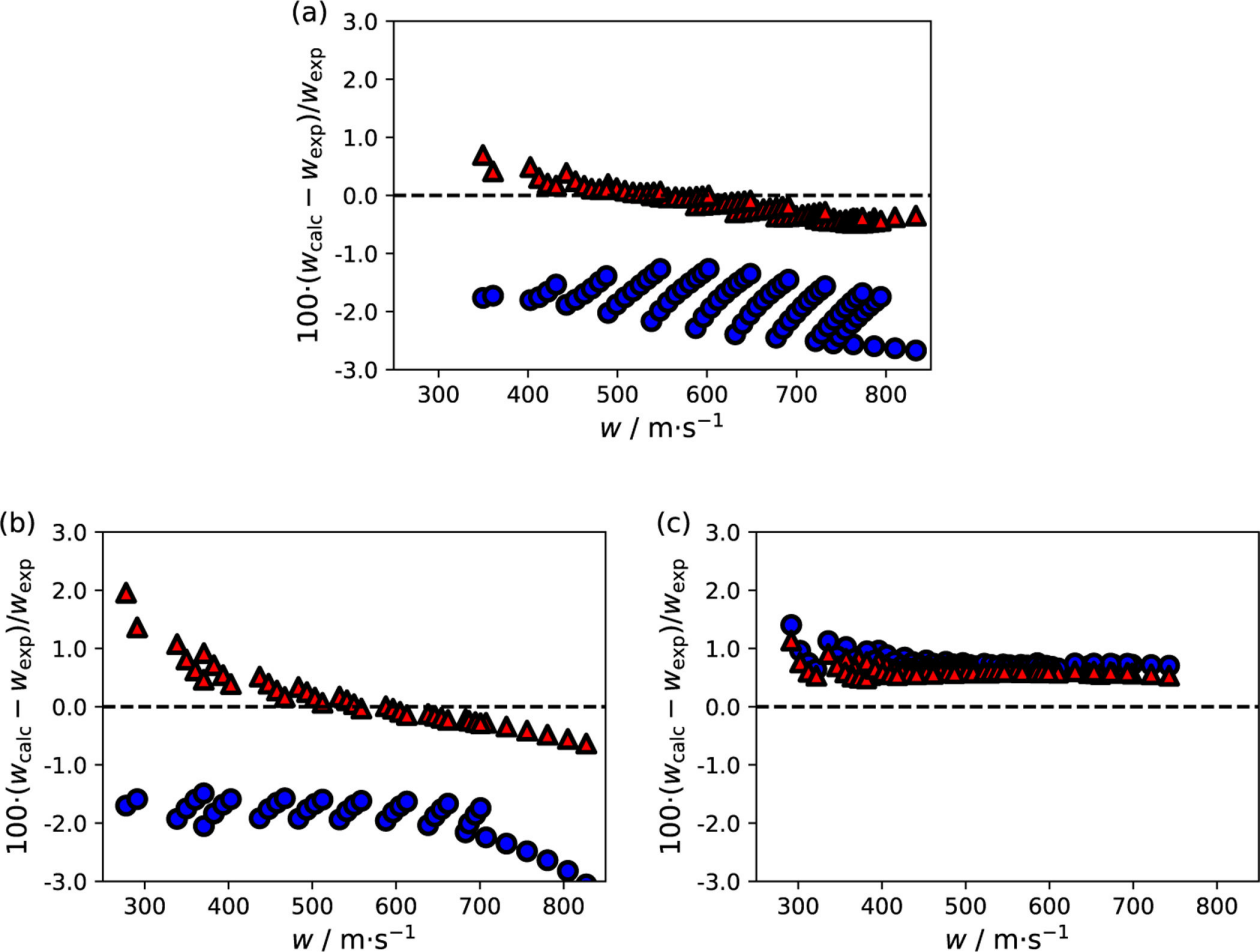
Deviation graphs comparing experimental speed of sound data, *w*_exp_, to speed of sound values calculated with REFPROP using the Helmholtz-energy-explicit EoS and the default binary interaction parameters used in REFPROP v10.0 [26] 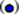 and updated parameters reported by Bell [15] 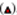, *w*_cal_, as a function of the exprimental speed of sound, *w*_exp_, where (a) R-444A, (b) R-457B, and (c) Ternary 4

Figure 3a-c are deviation plots comparing the experimental data reported in this study to both the default REFPROP v10.0 [26] models and those incorporating the updated binary interation parameters reported by Bell [14, 15]. The systematic deviations for the R-444A mixture and the R-457B mixture, which were formerly on the order of 2%, are removed, and remaining deviations are nearly all within 1%. It has been previously identified [15, 35] that the R-32 pure fluid EoS has deviations in speed of sound that could account for the remaining deviations.

**Fig. 4:**
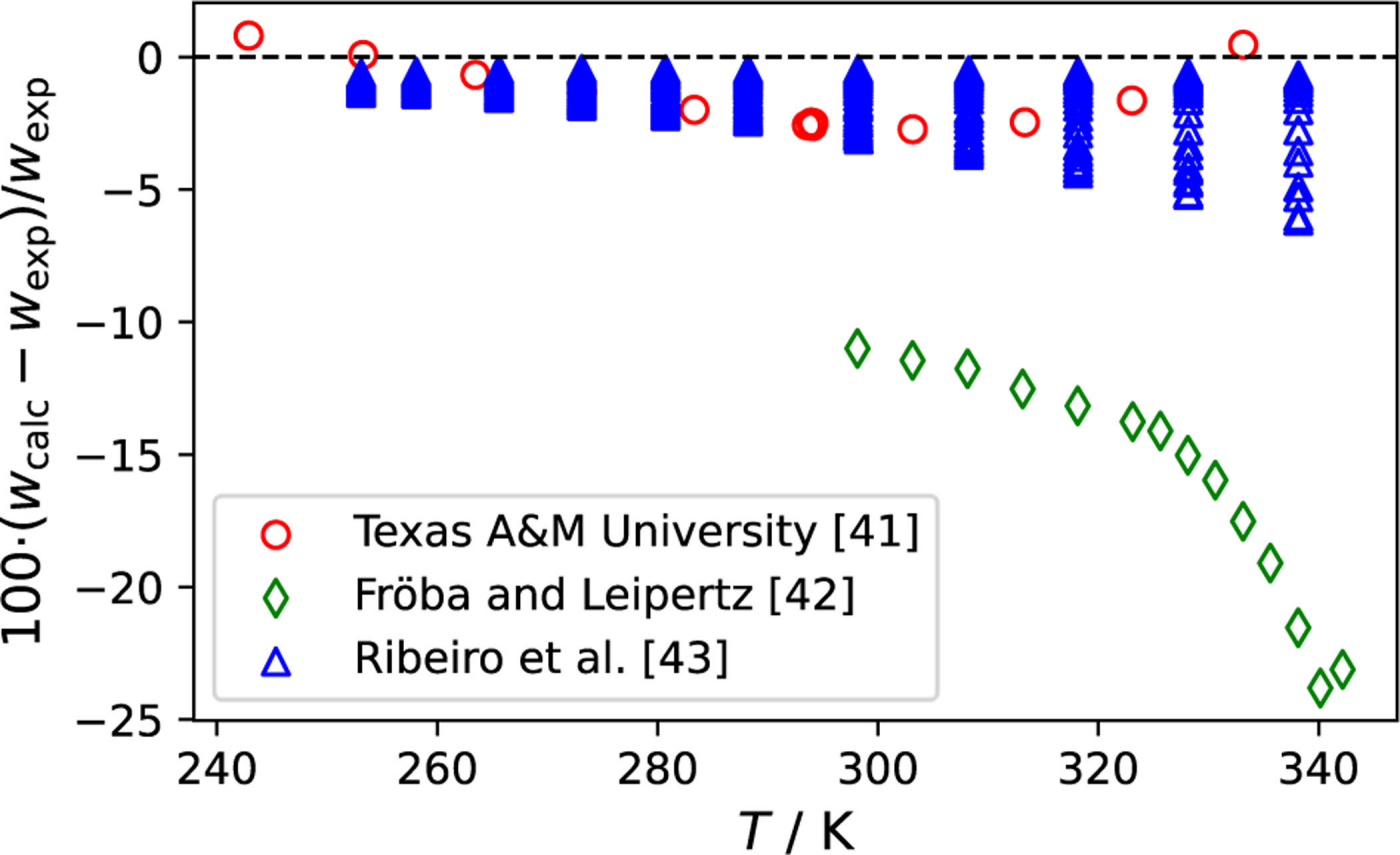
REFPROP v10.0 default model performance in speed of sound relative to available experimental data for the binary mixture R-125/32 [44–46]

The mixture R-407C contains only HFCs, and no HFOs, so the mixture model is still that of Lemmon and Jacobsen [39]. There is a mean bias error of – 0.23% compared to the experimental values. The mixture model used in Lemmon and Jacobsen [39] for the R-125/134a pair performed well when compared to unpublished vapor-phase speed of sound data ($\Delta_{\mathrm{AAD}}$ =0.020%) but did not include any speed of sound data in the fitting procedure. The binary of R-32/134a has one vapor-phase dataset [43] that is very well represented (within 0.03%) but no liquid phase data have been reported for this pair. The binary of R-125/32 is the most measured of the three, but the data selected when developing the model of Lemmon and Jacobsen do not appear to include liquid phase speed of sound. Figure 4 suggests that the deviations in the R-125/R-32 binary pair parameters likely contribute to the systematic deviation shown in Fig. 2.

**Fig. 5:**
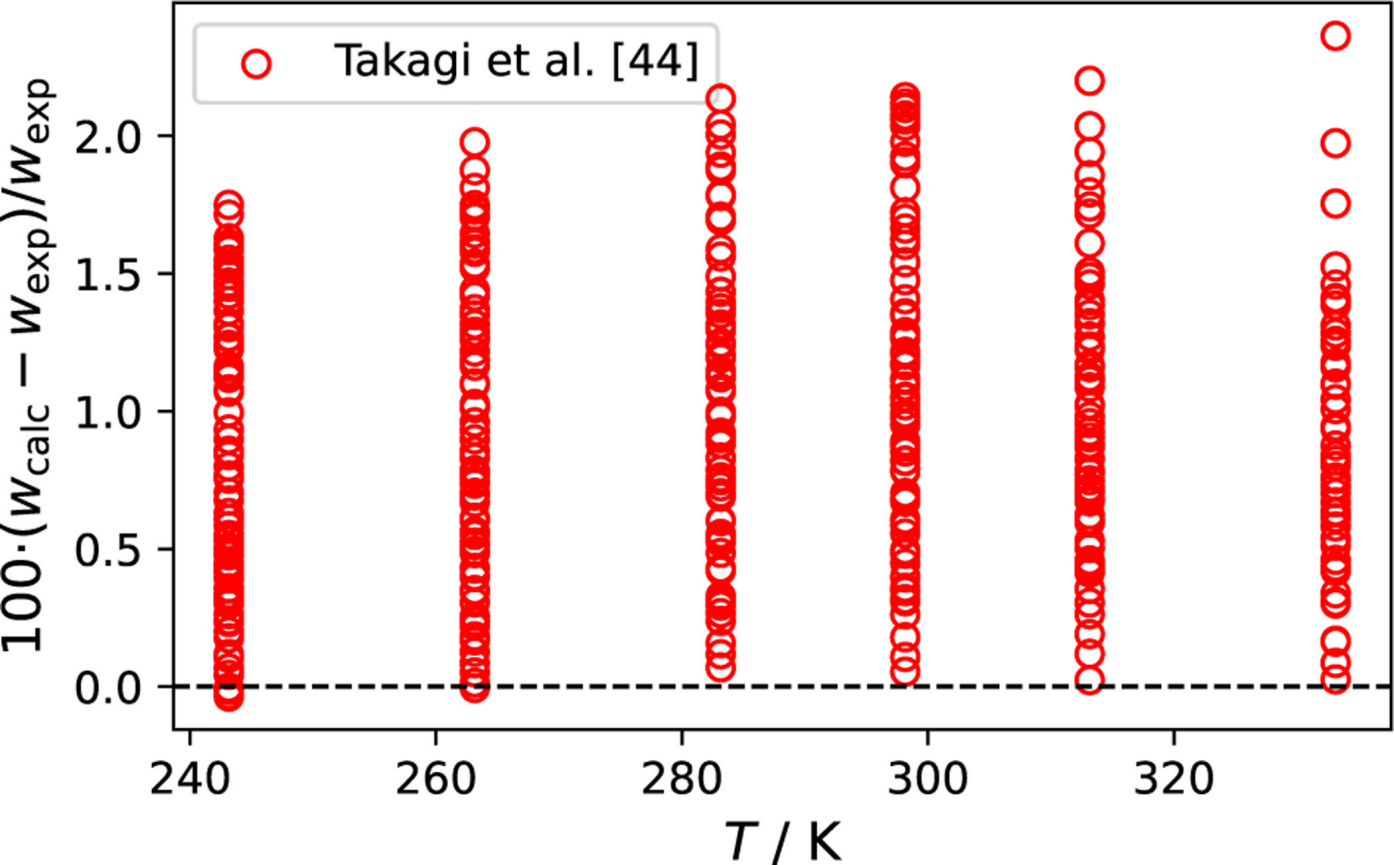
Deviation graph comparing experimental speed of sound data, *w*_exp_, for R-125/152a from Takagi et al. [47] to speed of sound values calculated with REFPROP v10.0 using the mixture model from Bell and Lemmon [42]

The deviations in Fig. 3 for the Ternary 4 blend also demonstrate a systematic offset. The Ternary 4 blend consists of the three components R-125, R-1234yf, and R-152a, so the binary mixture models for R-125/1234yf, R-1234yf/152a, and R-152a/125 are invoked. The mixture models for the pairs containing R-1234yf were included in Bell [14, 15] and the deviations in *w* were all within 0.5%. However, the R-125/R-152a pair uses the interaction parameters from Bell and Lemmon [42] which were fit only to VLE data. Sound speed for the R-125/R-152a pair was experimentally studied by Takagi et al. [47], and the deviations are shown in Fig. 5. The deviations for this mixture are characterized by a mean bias error of 0.95%. We believe this mixture model needs to be refit, and if one were to do so, the systematic offset in the ternary data would likely be largely removed.

## 4 Conclusions

The liquid phase speed of sound of several industrially relevant HFC/HFO ternary mixtures was measured using a dual-path pulse-echo technique. The data are reported for the refrigerant blends R-407C, R-444A, R-457B, and a custom blend designated as Ternary 4 (R-125/1234yf/152a) with mass compositions of (0.3521/0.5465/0.1014). Measurements were conducted at temperatures and pressures ranging between 230 K and 345 K and 0.14 MPa and 30 MPa, respectively. The combined expanded uncertainties of the measured speed of sound values ranged between 0.037% and 0.41%, with the largest deviations occuring near the critical points. The predictive capabilities of currently employed REFPROP v10.0 models for estimating ternary mixture speed of sound values are assessed. These models yield average deviations as high as 1.90% for the investigated mixtures, primarily due to weaknesses in several of the fitted binary interaction parameters which were mainly fit to VLE and/or density data. We quantified the improvements obtainable with updated binary interaction parameters that incorporated speed of sound data in the fitting procedure. Employing the updated binary interaction parameters reduces the average deviations in the predicted ternary values by 1.64% for R-444A and 1.50% for R-457B. Remaining deviations, which are less than 0.50%, are attributed largely to inaccuracies in the pure fluid EoS, mainly that of R-32. In contrast, the updated parameters yield only marginal improvements for the Ternary 4 blend. A more accurate prediction for this mixture is hampered by a mean bias error of 0.95% in the speed of sound of the R-125/152a binary pair. This work highlights the improvements achieved with updated HFC/HFO binary interaction parameters and an updated R-1234yf pure fluid EoS. Refitting the HFC/HFC interaction parameters to speed of sound data is expected to yield further improvements. Nevertheless, while the model accuracy improves when speed of sound data is included in the fitting, the mixture equations still perform reasonably well even when the mixing parameters are not fitted to speed of sound data. This indicates that (1) the current mixture model can be extended to additional properties not included in the fitting (albeit with higher uncertainties) and (2) ternary mixtures are well represented in terms of their constituent binary pairs.

## Supplementary Material

Supp1

**Supplementary Information** The online version contains supplementary material available at https://doi.org/10.1007/s10765-025-03608-3.

## Figures and Tables

**Table 1:** Refrigerant samples used in this study listed with their CAS numbers, molar mass (M), source, and purity

Chemical name and “R-number”	CAS number	M/g•mol^−1^	Source	Purity (Mole)

1,1,1,2-tetrafluoroethane (R-134a)	811–97–2	102.03	Dupont	99.9%
2,3,3,3-tetrafluoropropene (R-1234yf)	754–12–1	114.04	Chemours	99.99%
*trans* -1,3,3,3-tetrafluoropropene (R-1234ze(E))	29118–24–9	114.04	Honeywell	99.97%
Difluoromethane (R-32)	75–10–5	52.02	Advanced specialty gases	99.9%
Pentafluoroethane (R-125)	354–33–6	120.02	Scott specialty gas	99.99%
1,1-difluoroethane (R-152a)	75–37–6	66.05	Chemours	99.94%

All samples were degassed using a freeze-pump-thaw method prior to preparing mixtures

**Table 2:** Ternary mixture compositions and the combined standard uncertainties, *u*_c_(*x*_1_), of the compositions

Component 1 (Mass frac.)	Component 2 (Mass frac.)	Component 3 (Mass frac.)	ASHRAE name	*u*_c_(*x*_1_) (Mass frac.)

R-32 (0.5178)	R-125 (0.2480)	R-134a (0.2342)	R-407C	0.0005
R-32 (0.1194)	R-152a (0.0519)	R-1234ze(E) (0.8287)	R-444A	0.0003
R-32 (0.3489)	R-1234yf (0.5495)	R-152a (0.1016)	R-457B	0.0004
R-125 (0.3521)	R-1234yf (0.5465)	R-152a (0.1014)	Ternary 4^a^	0.0001

a Custom blend defined here with no ASHRAE identification

**Table 3:** Experimental speed of sound data for R-407C, a blend of R-32/125/134a with a mass composition of (0.5178/0.2480/0.2342)

*T*/K	*p*/MPa	*w*/m•s^−1^	100•*U*_r_(*w*)

249.998	1.160	715.70	0.045
254.992	2.569	701.75	0.046
259.992	6.955	709.47	0.044
264.982	11.292	716.79	0.043
269.978	15.636	724.05	0.042
274.973	20.072	731.78	0.041
279.976	24.492	739.28	0.040
284.972	28.842	746.31	0.040
289.976	33.202	753.29	0.039
259.989	2.123	674.44	0.050
264.981	6.278	682.26	0.048
269.976	10.455	690.07	0.047
274.970	14.630	697.74	0.044
279.976	18.792	705.17	0.043
284.971	22.947	712.45	0.042
289.977	27.101	719.62	0.041
294.966	31.219	726.57	0.040
299.966	35.334	733.36	0.039
269.976	2.084	625.62	0.057
274.969	5.882	633.96	0.053
279.975	9.718	642.31	0.051
284.970	13.519	650.21	0.049
289.975	17.336	658.03	0.049
294.965	21.134	665.61	0.045
299.966	24.902	672.82	0.044
304.977	28.668	679.86	0.042
309.974	32.402	686.68	0.042
279.974	2.337	578.92	0.064
284.969	5.821	587.92	0.060
289.976	9.321	596.69	0.056
294.965	12.790	604.96	0.053
299.966	16.276	613.06	0.051
304.977	19.751	620.88	0.049
309.974	23.208	628.40	0.048
314.973	26.653	635.68	0.046
319.973	30.083	642.73	0.045
289.974	1.779	522.67	0.079
294.963	4.872	532.24	0.073
299.965	7.990	541.50	0.069
304.976	11.111	550.37	0.064
309.974	14.215	558.81	0.060
314.973	17.329	567.01	0.057
319.973	20.431	574.90	0.054
324.982	23.532	582.51	0.051
329.975	26.615	589.85	0.050
299.964	1.999	472.93	0.103
304.975	4.748	482.85	0.089
309.972	7.531	492.59	0.081
314.973	10.322	501.89	0.075
319.973	13.110	510.70	0.070
324.982	15.897	519.10	0.065
329.976	18.668	527.12	0.062
334.978	21.379	534.39	0.059
339.974	24.114	541.63	0.056
309.970	2.245	421.63	0.130
314.970	4.692	432.60	0.113
319.971	7.140	442.70	0.101
324.982	9.599	452.26	0.092
329.975	12.056	461.31	0.085
334.975	14.461	469.38	0.079
339.973	16.890	477.36	0.074
344.986	19.339	485.19	0.070
349.992	21.783	492.74	0.066
319.969	3.008	377.39	0.166
324.979	5.167	388.77	0.143
329.973	7.305	398.81	0.126
334.977	9.384	407.36	0.114
339.972	11.529	416.29	0.104
344.986	13.694	424.95	0.095
349.992	15.862	433.25	0.088
329.973	3.062	315.48	0.271
334.974	4.818	327.30	0.223
339.972	6.614	338.40	0.188
344.985	8.436	348.80	0.163
349.992	10.267	358.53	0.145

Listed are the temperature, *T*, pressure, *p*, speed of sound, *w*, and relative combined expanded uncertainty of the speed of sound, *U*_r_(*w*) Speed of sound values listed are averaged from up to twelve measurements at each state point

* The combined standard uncertainty of the mass composition is *u*_c_(*x*_1_) = 0.0005, and the standard uncertainties for temperature and pressure are *u*_c_(*T*) = 0.005 K and *u*_*c*_*(p*) = 0.014 MPa, respectively. Expanded uncertainties are specified with a coverage factor, *k* = 2

**Table 4:** Experimental speed of sound data for R-444A, a blend of R-32/152a/1234ze(E) with a mass composition of (0.1194/0.0519/0.8287)

*T/K*	*p*/MPa	*w*/m•s^−1^	100•*U*_r_(*w*)

230.000	0.608	856.54	0.038
234.993	0.693	833.44	0.038
239.997	0.726	810.06	0.039
244.993	0.762	786.80	0.041
250.001	0.791	763.47	0.042
254.994	1.021	741.67	0.044
254.994	0.939	741.20	0.044
259.994	5.232	747.98	0.043
264.985	9.532	754.89	0.041
269.981	13.817	761.65	0.040
274.975	18.102	768.43	0.040
279.981	22.372	775.08	0.039
284.974	26.602	781.54	0.038
289.980	30.834	787.98	0.038
294.969	35.024	794.24	0.037
259.992	1.422	721.31	0.045
264.983	5.561	728.32	0.044
269.980	9.702	735.29	0.043
274.974	13.816	742.08	0.042
279.980	17.904	748.66	0.041
284.974	21.948	755.02	0.040
289.980	25.970	761.21	0.039
294.969	29.974	767.38	0.039
299.970	33.988	773.57	0.038
269.979	1.738	677.29	0.049
274.973	5.581	684.84	0.047
279.978	9.426	692.27	0.046
284.974	13.242	699.46	0.044
289.979	17.046	706.46	0.043
294.969	20.796	713.12	0.042
299.970	24.524	719.58	0.041
304.980	28.232	725.87	0.041
309.977	31.913	732.03	0.040
279.977	1.831	631.52	0.055
284.973	5.382	639.69	0.053
289.979	8.946	647.75	0.050
294.968	12.472	655.40	0.049
299.968	16.011	663.00	0.047
304.979	19.521	670.23	0.045
309.976	23.032	677.42	0.044
314.976	26.500	684.22	0.043
319.973	29.976	691.02	0.042
289.977	2.130	587.42	0.062
294.967	5.376	595.99	0.059
299.967	8.626	604.28	0.056
304.978	11.876	612.32	0.053
309.975	15.106	620.06	0.051
314.975	18.327	627.56	0.049
319.975	21.522	634.73	0.048
324.985	24.712	641.74	0.046
329.963	27.860	648.45	0.045
299.967	1.952	537.93	0.074
304.977	4.922	547.34	0.069
309.975	7.868	556.15	0.064
314.975	10.837	564.78	0.062
319.975	13.750	572.74	0.058
324.985	16.682	580.58	0.055
329.978	19.600	588.16	0.053
334.977	22.420	594.82	0.051
339.973	25.301	601.82	0.050
309.974	2.000	489.45	0.090
314.974	4.637	499.05	0.083
319.973	7.284	508.28	0.077
324.983	9.942	517.19	0.072
329.977	12.568	525.47	0.067
334.976	15.150	533.04	0.064
339.973	17.730	540.43	0.061
344.987	20.334	547.78	0.058
319.972	2.292	442.86	0.113
324.983	4.655	453.05	0.102
329.975	7.032	462.81	0.093
334.975	9.328	471.09	0.087
339.974	11.689	479.73	0.081
344.986	14.022	487.72	0.076
329.975	3.015	402.56	0.142
334.975	5.077	412.29	0.125
339.974	7.176	421.92	0.113
344.986	9.311	431.37	0.104
339.973	3.113	349.61	0.199
344.987	4.939	360.91	0.172

Listed are the temperature, *T,* pressure, *p*, speed of sound, *w*, and relative combined expanded uncertainty of the speed of sound, *U*_r_(*w*)

Speed of sound values listed are averaged from up to twelve measurements at each state point

* The combined standard uncertainty of the mass composition is *u*_c_(*x*_1_) = 0.0003, and the standard uncertainties for temperature and pressure are *u*_c_(*T*) = 0.005 K and *u*_*c*_(*p*) = 0.014 MPa, respectively. Expanded uncertainties are specified with a coverage factor, *k* = 2

**Table 5:** Experimental speed of sound data for R-457B, a blend of R-32/1234yf/152a with a mass composition of (0.3489/0.5495/0.1016)

*T*/K	*p*/MPa	*w*/m•s^−1^	100•*U*_r_(*w*)

230.000	0.715	827.02	0.040
234.994	1.135	805.00	0.041
239.998	1.190	780.70	0.042
244.993	1.222	756.28	0.044
250.001	1.254	731.77	0.046
254.994	1.293	707.35	0.048
259.995	1.344	682.94	0.050
264.985	4.726	686.94	0.049
269.982	8.582	694.66	0.047
274.975	12.232	700.78	0.046
269.979	1.891	638.17	0.056
274.976	5.428	646.19	0.053
279.980	8.975	654.08	0.051
284.975	12.533	661.94	0.049
279.979	1.891	587.72	0.065
284.975	5.105	596.29	0.061
289.980	8.337	604.72	0.058
294.969	11.557	612.88	0.055
289.979	1.617	532.44	0.081
294.970	4.457	541.29	0.075
299.970	7.299	549.74	0.070
304.980	10.199	558.47	0.065
299.969	2.003	483.46	0.101
304.979	4.607	493.54	0.091
309.977	7.210	503.09	0.083
314.976	9.814	512.17	0.077
309.976	2.634	437.11	0.128
314.977	4.952	447.72	0.113
319.976	7.277	457.68	0.102
324.985	9.611	467.12	0.093
319.975	2.363	370.54	0.201
324.986	4.330	382.49	0.171
329.979	6.302	393.44	0.148
334.977	8.227	402.72	0.132
329.978	3.743	338.51	0.242
334.977	5.460	349.65	0.205
339.975	7.210	360.29	0.177
344.987	8.978	370.32	0.155
339.974	4.073	277.61	0.412
344.987	5.510	290.87	0.327

Listed are the temperature, *T*, pressure, *p*, speed of sound, *w*, and relative combined expanded uncertainty of the speed of sound, *U*_r_(*w*) Speed of sound values listed are averaged from up to twelve measurements at each state point

* The combined standard uncertainty of the mass composition is *u*_c_(*x*_1_) = 0.0004, and the standard uncertainties for temperature and pressure are *u*_c_(*T*) = 0.005 K and *u*_*c*_*(p*) = 0.014 MPa, respectively. Expanded uncertainties are specified with a coverage factor, *k* = 2

**Table 6:** Experimental speed of sound data for the R-125/1234yf/152a, Ternary 4, mixture with a mass composition of (0.3521/0.5465/0.1014)

*T*/K	*p*/MPa	*w*/m•s^−1^	100•*U*_r_(*w*)

230.001	0.603	742.73	0.042
234.995	0.878	721.46	0.044
239.998	0.909	698.72	0.045
244.993	0.917	675.94	0.048
250.001	0.967	653.46	0.050
254.995	2.960	647.07	0.050
259.996	6.619	654.01	0.048
264.986	10.251	660.75	0.047
264.985	1.324	588.60	0.060
269.982	4.570	595.82	0.056
274.975	7.823	603.02	0.054
279.981	11.116	610.38	0.052
269.981	1.321	565.89	0.064
274.975	4.426	573.42	0.060
279.981	7.559	581.00	0.057
284.975	10.683	588.41	0.055
274.975	1.477	544.71	0.068
279.980	4.449	552.46	0.064
284.976	7.423	560.10	0.061
289.982	10.382	567.40	0.058
279.980	1.499	522.10	0.075
284.975	4.325	530.09	0.070
289.982	7.173	538.04	0.065
294.971	10.012	545.73	0.062
284.975	1.290	496.62	0.082
289.981	3.943	504.68	0.077
294.971	6.616	512.70	0.072
299.972	9.326	520.81	0.068
304.982	12.023	528.52	0.064
289.979	1.539	476.26	0.089
294.969	4.084	484.81	0.083
299.971	6.647	493.14	0.077
304.981	9.225	501.30	0.073
309.979	11.799	509.17	0.068
294.968	1.544	452.82	0.100
299.970	3.939	461.43	0.092
304.981	6.381	470.20	0.086
309.978	8.819	478.55	0.079
314.977	11.254	486.56	0.075
299.969	1.44	427.24	0.114
304.980	3.719	436.49	0.104
309.977	6.004	445.42	0.095
314.977	8.300	454.01	0.088
319.977	10.592	462.20	0.082
304.979	1.061	396.01	0.136
309.977	1.203	372.48	0.158
314.977	1.783	357.22	0.172
319.977	3.673	367.66	0.153
324.988	5.567	377.29	0.137
329.979	7.471	386.48	0.124
319.977	2.049	335.90	0.199
324.986	3.812	346.48	0.175
329.980	5.576	356.22	0.156
334.979	7.308	364.78	0.141
339.976	9.060	373.10	0.129
344.989	10.835	381.27	0.118
329.978	2.542	291.71	0.279
334.977	4.030	302.34	0.241
339.974	5.546	312.36	0.210
344.987	7.074	321.67	0.186
239.996	0.139	693.04	0.046
244.991	0.528	672.89	0.048
249.999	0.918	653.02	0.050
254.993	0.937	630.41	0.053
264.985	1.004	585.45	0.061
269.982	3.266	584.19	0.059
274.975	6.443	591.37	0.056
279.980	9.653	598.67	0.054
279.980	1.510	522.18	0.074
284.975	4.334	530.18	0.070
289.981	7.190	538.20	0.065
294.970	10.036	545.96	0.062
299.971	1.389	426.48	0.114
304.980	3.667	435.86	0.105
309.977	5.947	444.75	0.096
314.977	8.219	453.10	0.089
319.976	10.488	461.11	0.083
304.978	1.631	405.53	0.128
309.975	3.778	415.10	0.116
314.976	5.942	424.28	0.105
319.976	8.105	432.96	0.097
324.985	10.276	441.29	0.090
309.975	1.725	382.01	0.147
314.975	3.739	391.89	0.132
319.976	5.753	401.07	0.119
324.986	7.783	409.90	0.109
329.978	9.807	418.25	0.100

Listed are the temperature, *T,* pressure, *p*, speed of sound, *w*, and relative combined expanded uncertainty of the speed of sound, *U*_r_(*w*)

Speed of sound values listed are averaged from up to twelve measurements at each state point

* The combined standard uncertainty of the mass composition is *u*_c_(*x*_1_) = 0.0001, and the standard uncertainties for temperature and pressure are *u*_c_(*T*) = 0.005 K and *u*_*c*_*(p*) = 0.014 MPa, respectively. Expanded uncertainties are specified with a coverage factor, *k* = 2

**Table 7:** Summary of model comparisons to the data listed in Tables 3 through 6 for the R-407C, R-444A, R-457B, and Ternary 4 mixtures

Mixture	Pure fluid EoS	Binary interaction parameters	$\Delta_{\mathrm{AAD}}$/ %	$\Delta_{\mathrm{MAX}}$/ %

Default REFPROP Version 10.0 mixture models [26]				
R-407C	R-32 [32] R-125 [38] R-134a [10]	R-32/125 [39] R-32/134a [39] R-125/134a [39]	0.23	0.78
R-444A	R-32 [32] R-152a [40] R-1234ze(E) [31]	R-32/152a^a^ R-32/1234ze(E) [41] R-152a/1234ze(E) [42]^b^	1.89	2.71
R-457B	R-32 [32] R-1234yf [36] R-152a [40]	R-32/1234yf [41] R-32/152a^a^ R-1234yf/152a [42]	1.90	3.05
Ternary 4	R-125 [38] R-1234yf [36] R-152a [40]	R-125/1234yf^a^ R-125/152a [42] R-1234yf/152a [42]	0.73	1.40
Models with updated binary interaction parameters [15]				
R-444A	R-32 [32] R-152a [40] R-1234ze(E) [31]	R-32/152a^a^ R-32/1234ze(E) [15] R-152a/1234ze(E) [42]^b^	0.25	0.70
R-457B	R-32 [32] R-1234yf [37] R-152a [40]	R-32/1234yf [15] R-32/152a^a^ R-1234yf/152a [15]	0.40	1.96
Ternary 4	R-125 [38] R-1234yf [37] R-152a [40]	R-125/1234yf [15] R-125/152a [42] R-1234yf/152a [15]	0.62	1.14

a Parameters included in REFPROP v10.0 and not included in a journal publication

b Parameters estimated using REFPROP v10.0 based on a chemically similar system

## Data Availability

Data listing all the unaveraged speed of sound measurements and their associated uncertainties are deposited at nist.data.gov (https://doi.org/10.18434/mds2-3874).
