# Supplementary material for: Speed of Sound Measurements of Select Ternary Refrigerant Mixtures and Predictions Using Constituent Binary Interaction Parameters
Source: Int J Thermophys. Author manuscript; Available in PMC 2026 Jul 9. (PMC13344128; doi:10.1007/s10765-025-03608-3)
Supplement: Supp1 [file NIHMS2113867-supplement-Supp1.docx]

Supplementary Information

**Speed of Sound Measurements of Select Ternary Refrigerant Mixtures and Predictions using Constituent Binary Interaction Parameters**

Karim S. Al-Barghouti^1^, Katrina N. Avery^1^, Ian H. Bell^1^, and Aaron J. Rowane^1,^*

^1^Applied Chemicals and Materials Division, National Institute of Standards and Technology, Boulder, Colorado 80305, United States

* Corresponding author Email: Aaron.Rowane@nist.gov

Contents contained in Supplementary Information

- Speed of sound experimental data plots for R-407C, R-457B, and Ternary 4
- Raw speed of sound data from all trials for the R-407C, R-444A, R-457B, and Ternary 4 blends in a machine-readable format

**S1. Experimental Speed of Sound Plots**

Figure 1 of the main work includes a pseudo-isochore plot for R-444A illustrating speed of sound as a function of temperature and pressure. Similar plots are provided here for R-407C, R-457B, and Ternary 4 in figures S1, S2, and S3, respectively. The different symbols in the plots represent the different pseudo-isochores.


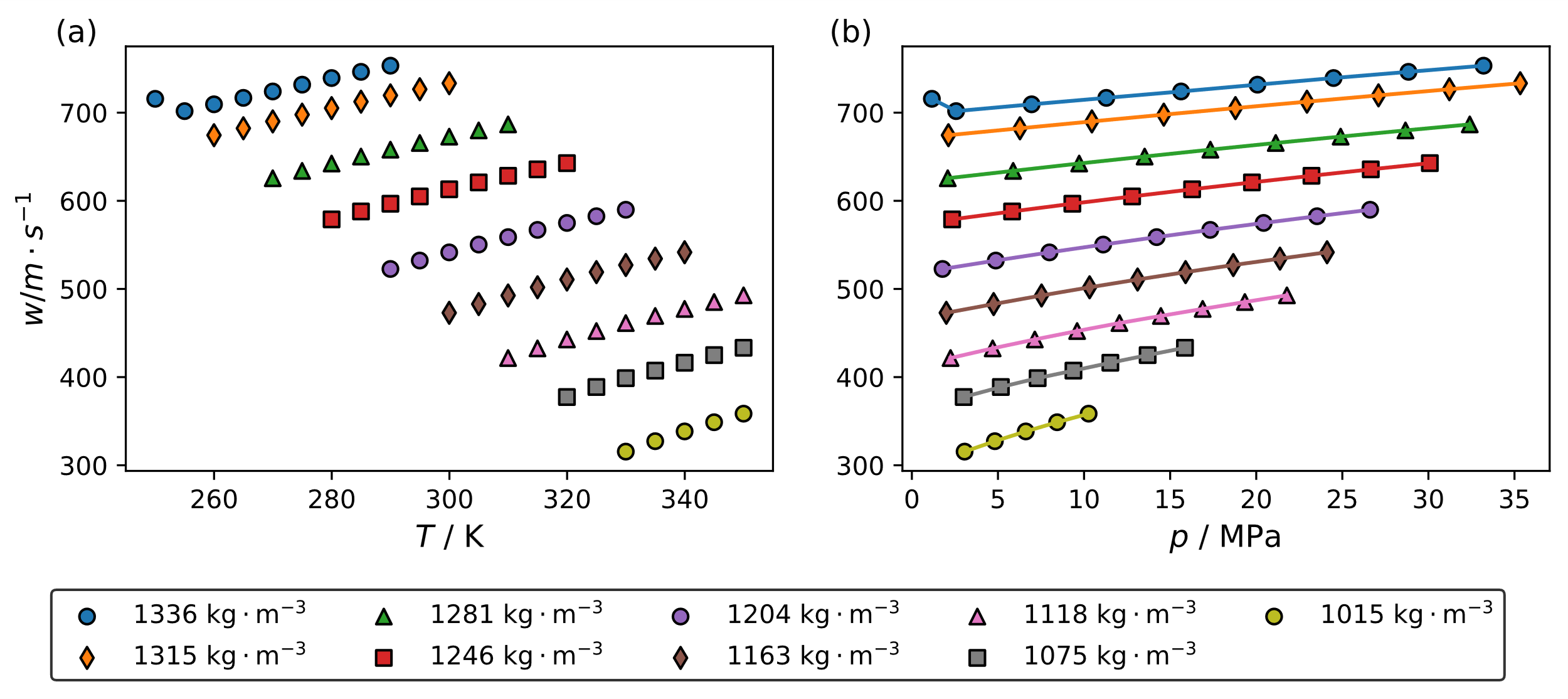


Figure S1. The effect of (a) temperature and (b) pressure on the speed of sound of R-407C. Different symbols in the legend represent the different pseudo-isochores measured. Lines are visual guides indicating data from the same isochore. The densities for each isochore are average densities across all state points for a given isochore, which were calculated using the latest EoS[1-3] for each component and the updated binary interaction parameters reported by Bell[4, 5].


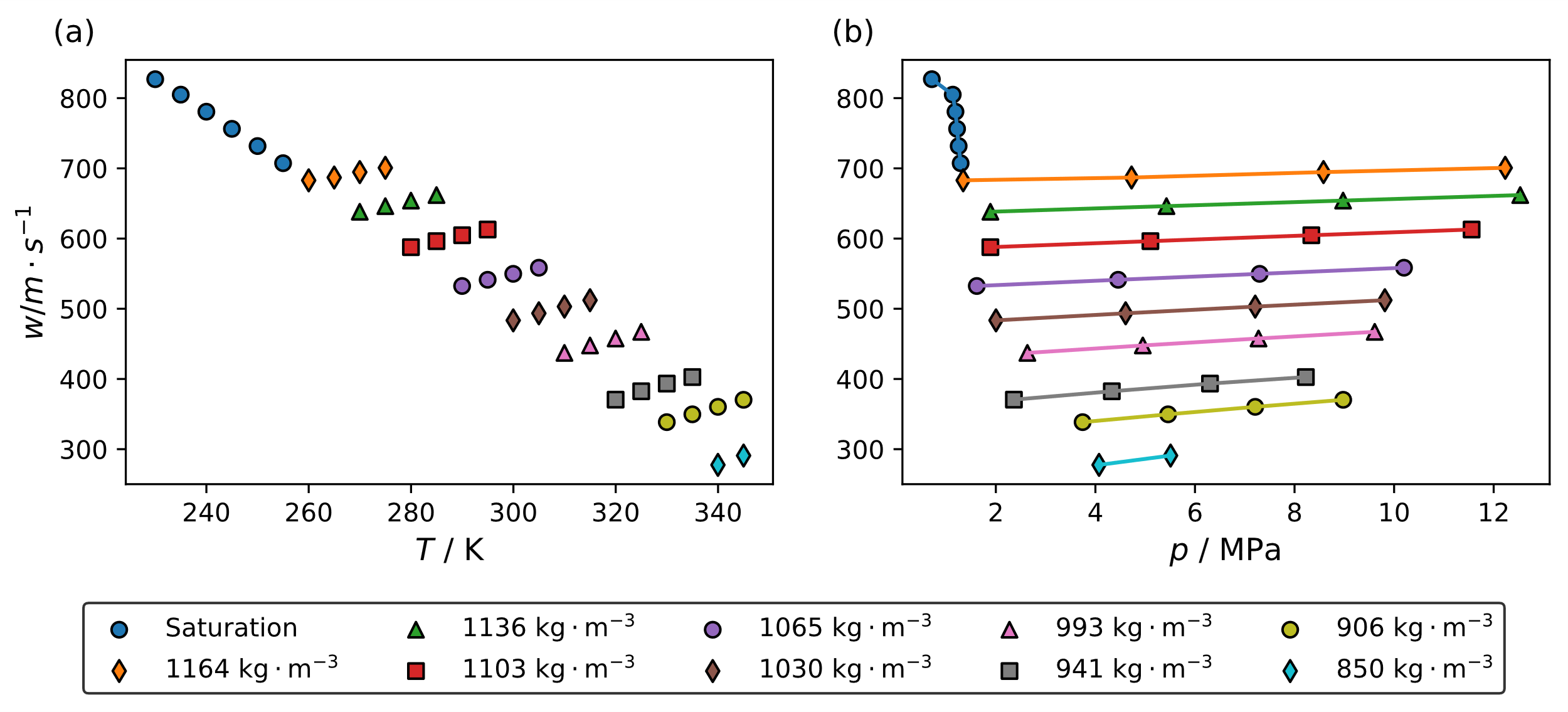


Figure S2. The effect of (a) temperature and (b) pressure on the speed of sound of R-457B. Different symbols in the legend represent the different pseudo-isochores measured. Lines are visual guides indicating data from the same isochore. The densities for each isochore are average densities across all state points for a given isochore, which were calculated using the latest EoS[1, 6, 7] for each component and the updated binary interaction parameters reported by Bell[4, 5].


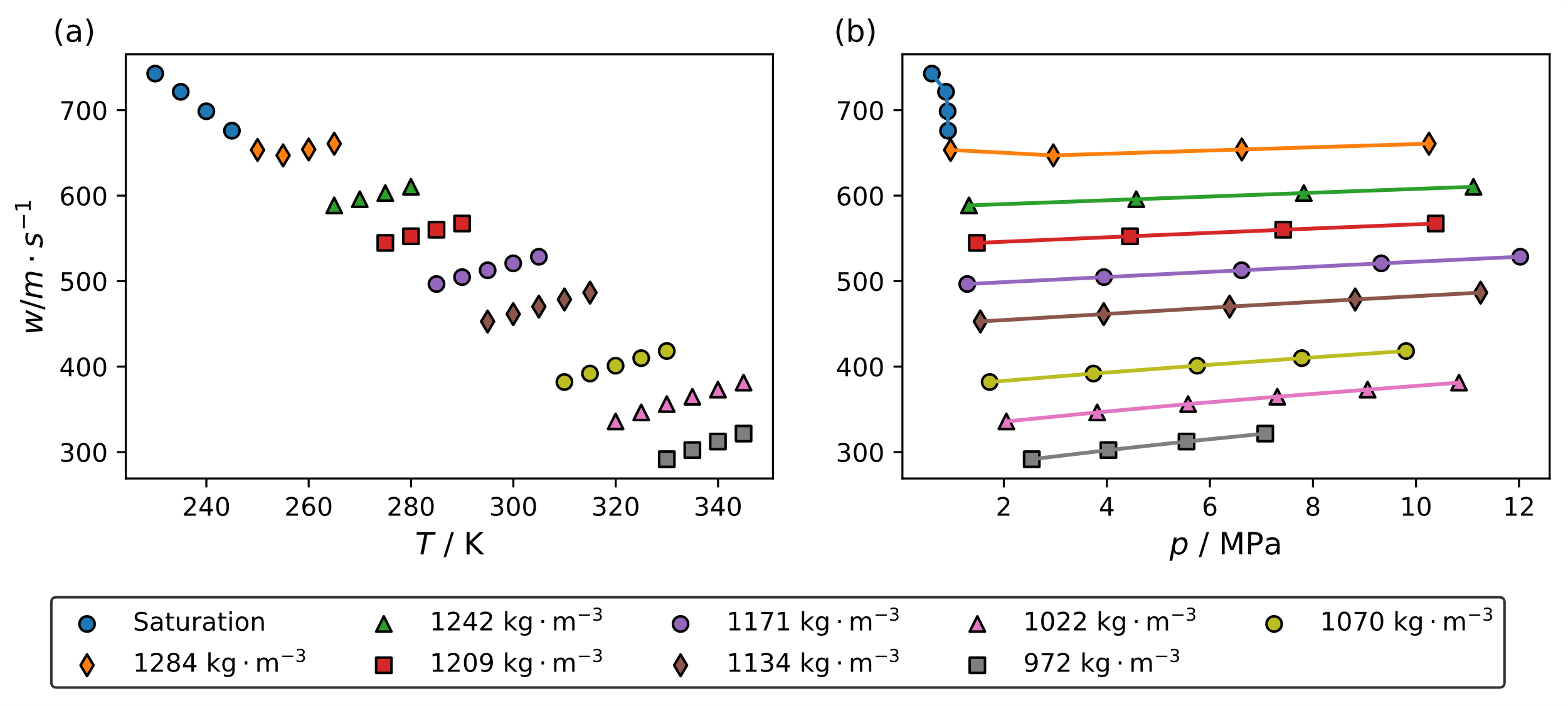


Figure S3. The effect of (a) temperature and (b) pressure on the speed of sound of Ternary 4. Different symbols in the legend represent the different pseudo-isochores measured. Lines are visual guides indicating data from the same isochore. The densities for each isochore are average densities across all state points for a given isochore, which were calculated using the latest EoS[2, 6, 7] for each component and the updated binary interaction parameters reported by Bell[4, 5].

References

1. R. Tillner-Roth, A. Yokozeki. An International Standard Equation of State for Difluoromethane (R-32) for Temperatures from the Triple Point at 136.34 K to 435 K and Pressures up to 70 MPa. *J. Phys. Chem. Ref. Data* **1997**, *26*, 1273. DOI: <https://doi.org/10.1063/1.556002>.

2. E.W. Lemmon, R.T. Jacobsen. A New Functional Form and New Fitting Techniques for Equations of State with Application to Pentafuloroethane (R-125). *J. Phys. Chem. Ref. Data* **2005**, *34*, 69. DOI: <https://doi.org/10.1063/1.1797813>.

3. R. Tillner-Roth, H.D. Baehr. An International Standard Formulation for the Thermodynamic Properties of 1,1,1,2-Tetrafluoroethane (HFC-134a) for Temperatures from 170 K to 455 K and Pressures up to 70 MPa. *J. Phys. Chem. Ref. Data* **1994**, *23* (5), 657. DOI: <https://dx.doi.org/10.1063/1.555958>.

4. I.H. Bell. Mixture Models for Refrigerants R-1234yf/134a, R-1234yf/1234ze(E), and R-134a/1234ze(E) and Interim Models for R-125/1234yf, R-1234ze(E)/227ea, and R-1234yf/152a. *J. Phys. Chem. Ref. Data* **2022**, *51* (1), 013103. DOI: <https://dx.doi.org/10.1063/5.0086060>.

5. I.H. Bell. Mixture Model for Refrigerant Pairs R-32/1234yf, R-32/1234ze(E), R-1234ze(E)/227ea, R-1234yf/152a, and R-125/1234yf. *J. Phys. Chem. Ref. Data* **2023**, *52* (1), 013101. DOI: <https://dx.doi.org/10.1063/5.0135368>.

6. S.L. Outcalt, M.O. McLinden. A Modified Benedict–Webb–Rubin Equation of State for the Thermodynamic Properties of R152a (1,1-difluoroethane). *J. Phys. Chem. Ref. Data* **1996**, *25* (2), 605. DOI: 10.1063/1.555979.

7. M. Richter, M.O. McLinden, E.W. Lemmon. Thermodynamic Properties of 2,3,3,3-Tetrafluoroprop-1-ene (R1234yf): Vapor Pressure and *p*-*ρ*-*T* Measurements and an Equation of State. *Journal of Chemical & Engineering Data* **2011**, *56*, 3254. DOI: <https://doi.org/10.1021/je200369m>.
